# Maternal high calorie diet induces mitochondrial dysfunction and senescence phenotype in subcutaneous fat of newborn mice

**DOI:** 10.18632/oncotarget.19948

**Published:** 2017-08-04

**Authors:** Daniele Lettieri-Barbato, Fabiana D’Angelo, Francesca Sciarretta, Giuseppe Tatulli, Flavia Tortolici, Maria Rosa Ciriolo, Katia Aquilano

**Affiliations:** ^1^ Department of Biology, University of Rome Tor Vergata, Rome, Italy; ^2^ IRCCS San Raffaele Pisana, Rome, Italy

**Keywords:** aging, senescence, adipocytes, inflammation, NAD, Gerotarget

## Abstract

Mitochondrial dysfunction, inflammation and senescence-like features are observed in adipose depots in aging and obesity. Herein, we evaluated how maternal high calorie diet (HCD) may impact on subcutaneous adipose tissue (sAT) of the newborn mice. Adult C57BL/6J mice were randomly divided in three groups: normal calorie diet (NCD), HCD and HCD supplemented with niacin 8 weeks before mating. Mothers and pups were then sacrificed and metabolic and molecular analyses were carried out on sAT. HCD induced mitochondria dysfunction in mothers without inflammation and senescence, whereas in pups we also revealed the occurrence of senescent phenotype. The mitochondrial dysfunction-associated senescence in pups was accompanied by a drop in NAD^+^/NADH ratio and alteration in the NAD^+^-dependent enzymes PARP1 and SIRT1. Importantly, maternal dietary supplementation with niacin during gestation and lactation restrained NAD^+^/NADH decrease imposed by HCD limiting inflammatory cytokine production and senescence phenotype in newborn sAT. Given the fundamental role of sAT in buffering nutrient overload and avoiding pathogenic ectopic fat accumulation, we suggest that NAD^+^ boosting strategies during maternal HCD could be helpful in limiting sAT dysfunction in newborn.

## INTRODUCTION

White adipose tissue has heterogeneous anatomic distribution in humans and can be divided into visceral (vAT) and subcutaneous (sAT) adipose tissue [1]. vAT and sAT differently develop during life and, after middle age, fat redistributes from subcutaneous to visceral depots [2]. White adipose depots have the function to directly buffer dietary fats thus limiting systemic lipotoxicity and ectopic fat accumulation [3]. However, vAT and sAT are different in terms of adipocyte dimension and metabolic activity [4, 5] as they differently respond to insulin and catecholamines. Specifically, vAT is more sensitive to lipolysis induced by catecholamines and less sensitive to the antilipolytic effect of insulin than is sAT. This features vAT as the most pathogenic and sAT as the least metabolically harmful storage site of dietary fat excess. There is common consensus that enlargement of vAT increases the risk of developing age-related metabolic dysfunctions including type 2 diabetes and is more strongly correlated to such disease states than sAT. On the contrary, retention of a high ratio of functioning sAT to vAT is associated with increased health span and longevity [6].

Aging as well as chronic overnutrition dictates the pathological expansion of AT that consists in the acquirement of a senescence-associated secretory phenotype (SASP) that leads to sterile inflammation and insulin resistance [7]. Mitochondria manage nutrient flux by producing energy or heat and are able to modulate intracellular redox pressure. When nutrients are in excess, mitochondria may undergo indecision in the selection of the substrates thus failing in properly adjusting their metabolic activity [8]. Furthermore, a massive mitochondrial electron transport chain activity and oxidative damage could be operative thus leading to the exhaustion of the metabolic capacity of cells such as adipocytes [6]. Recently, it has been demonstrated that the decrease of NAD^+^, occurring during aging or upon nutrient excess is the triggering factor of mitochondrial dysfunction and acquirement of a senescent and secretory phenotype in many cells and tissues [9, 10]. Poly(ADP-ribose) polymerase 1 (PARP1) and SIRT1 deacetylase are two NAD^+^-dependent enzymes that play a key role in the control of damage repair and metabolic homeostasis [11]. Given the dependence of PARP1 and SIRT1 activity on NAD^+^, mitochondria dysfunction could hence affect their activity.

Developmental programming in response to maternal overnutrition during gestation and lactation results in health span reduction in the offspring [12]. Retrospective case control studies found higher adiposity and metabolic defects and an increased risk of mortality in the offspring born from obese mothers compared to non-obese mothers [13, 14]. Maternal ovenutrition increases inflammatory cytokines expression in sAT of foetus and profoundly impacts on mitochondrial mass and functionality in brown adipose tissue of newborn mice [15, 16].

Herein we demonstrated that a high calorie diet (HCD), characterized by an excess of lipids during gestation and lactation, causes mitochondrial dysfunction in sAT of mothers and offspring pups. However, NAD^+^ depletion and the senescent phenotype were detected only in pups and these events were accompanied by affected SIRT1-mediated FoxO1 deacetylation and PARP1 activity. Avoiding NAD^+^ drop *via* dietary supplementation with niacin, even though unable to recover mitochondrial function, limited the occurrence of SASP through the restoring of PARP1 and SIRT1 activity. These findings suggest that, bypassing mitochondrial dysfunction and directly providing NAD^+^ at nuclear level, niacin might protect subcutaneous adipose tissue in pups preventing maternally-derived metabolic diseases.

## RESULTS

### High calorie diet induces mitochondrial rearrangement typical of aging in mouse sAT

**Figure 1:**
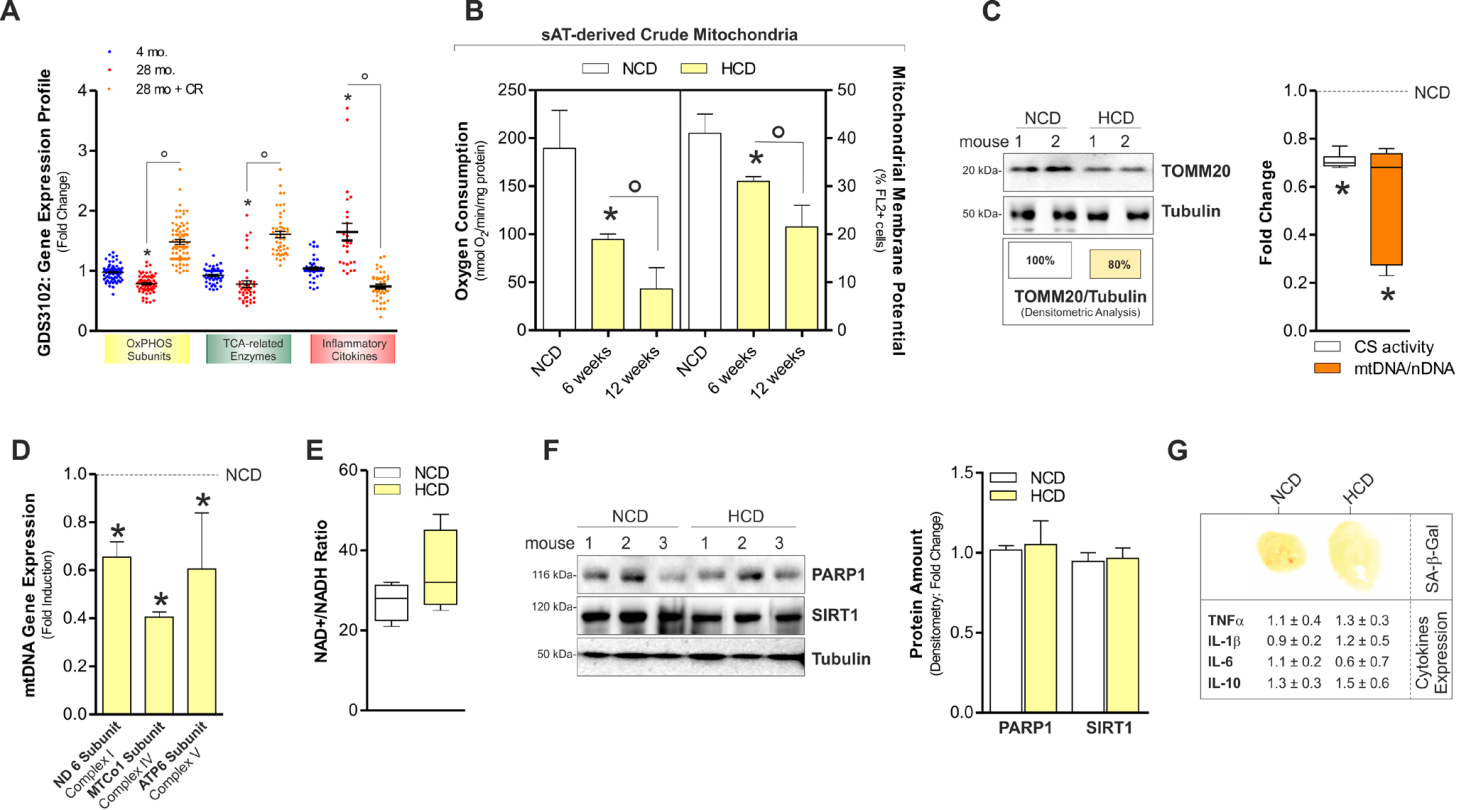
HCD induced mitochondrial dysfunction in on sAT of adult C57BL/6J female mice **A.** Analyses of data from the publicly available GEO data set (ID: GDS3102) revealed a down-regulation of mitochondrial OxPHOS subunits and TCA enzymes in the sAT of old (28 mo.) *versus* young (4 mo.) rats. Oppositely, inflammatory cytokines levels were higher in old than young rats. Calorie restriction (CR) significantly improved OxPHOS subunits and inflammatory cytokines gene transcription. Gene expression profile was obtained calculating the fold change of gene expression profile provided by gene expression omnibus (ID: GDS3102). For details related to gene included in our analyses, see Experimental Procedures section. **B.** Oxygen consumption and mitochondrial membrane potential measured in sAT-derived crude mitochondria of adult female mice 6 and 12 weeks after normal calorie diet (NCD) and high calorie diet (HCD). **C.** Mitochondrial mass evaluated by analysing the protein levels of mitochondrial TOMM20 in total sAT homogenates 12 weeks after NCD and HCD. Boxes reported below show the percentage of TOMM20 protein in total fraction (*left panel*). Fold change in citrate synthase (CS) activity and mtDNA/nDNA ratio detected in sAT 12 weeks after NCD and HCD (*right panel*). **D.** Measurement of the mRNA expression of mtDNA-encoded genes in sAT 12 weeks after NCD and HCD. **E.** NAD^+^/NADH ratio measured in total homogenates of sAT of mothers 12 weeks after NCD and HCD. **F.** PARP1 and SIRT1 protein levels and their densitometric analyses (*right panel*) in total homogenates of sAT 12 weeks after NCD and HCD. **G.** Senescence-associated β-galactosidase activity (*upper panel*) and mRNA expression of inflammatory cytokines (*lower panel*) in sAT 12 weeks after NCD and HCD. Tubulin served as loading control. All data are expressed as mean ±S.D. (*n* = 4 female adult mice/group; *p < 0.05 *vs* 4 mo. or NCD; °*p* < 0.05 *vs* 28 mo. or 6 weeks).

We previously demonstrated that lowering calorie intake through one-day fasting enhanced the expression of mitochondrial genes in sAT [17]. To more strictly establish whether the expression of mitochondrial genes varies in response to calorie intake and age in sAT, we moved at searching publically available gene expression data sets. We found a data set obtained by analysing sAT of young (~4 months) and aged (~28 months) rats subject to calorie restriction [18]. Gene set enrichment analysis [GSEA; Gene Expression Omnibus (GEO) accession numbers GDS3102] was expressed as fold change. As reported in Figure 1A, aged sAT displays a significant down-regulation of mitochondrial OxPHOS subunits and TCA-related enzymes according to the idea that mitochondrial efficiency declines during aging. Interestingly, calorie restriction reverts the decline of OxPHOS and TCA gene transcription occurring during aging. Hence, we attempted at evaluating the effects of HCD on sAT mitochondria and found a progressive drop of oxygen consumption and mitochondrial transmembrane potential (Figure 1B). Furthermore, HCD affected the level of the mitochondrial translocase TOMM20, mtDNA and citrate synthase activity (Figure 1C) as well as mRNA expression of mtDNA-encoded genes (Figure 1D), indicating a reduced mitochondrial mass and mitochondrial transcriptional activity of sAT. NAD+/NADH levels remained unaffected (Figure 1E) and accordingly the protein levels of the main NAD^+^-dependent enzymes SIRT1 and PARP1 were unchanged in sAT (Figure 1F).

Dysfunctional mitochondria have recently been associated with senescent phenotype in adipose tissue [9]. However, we did not reveal any senescence marker in sAT during HCD. Indeed, senescence-associated beta galactosidase activity (SA-β-Gal) was not detected and inflammatory cytokines expression (TNFα, IL-1β, IL-6, IL-10) was unaffected (Figure 1G). The absence of a senescent phenotype was confirmed by the analysis of the senescence markers cyclin-dependent kinase inhibitor 1 (p21^Waf1^; also known as CDKN1A) and phosphorylated histone H2A.X (H2A.Xp) that resulted undetectable (data not shown).

### High calorie diet during gestation and lactation induces mitochondrial dysfunction and senescence in subcutaneous adipose tissue of newborn mice

**Figure 2:**
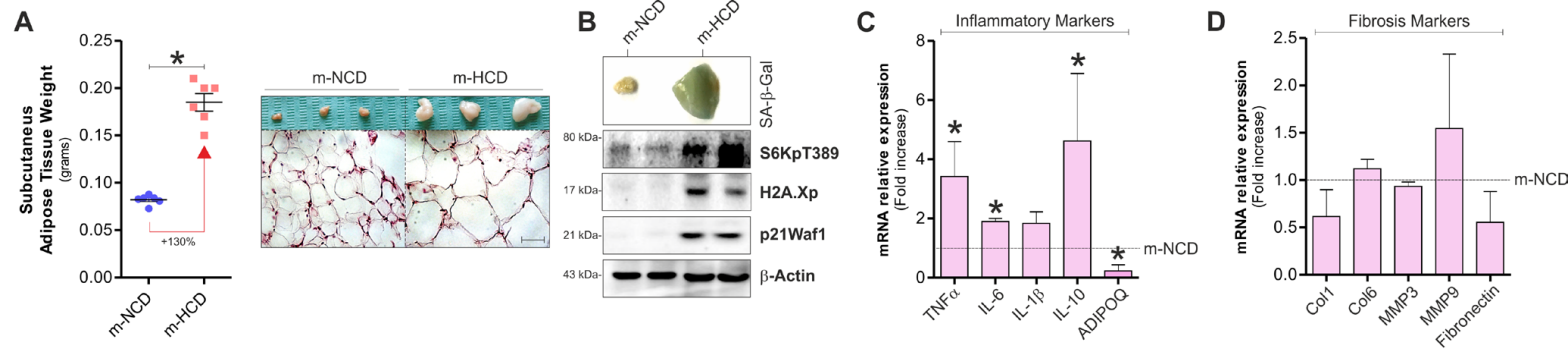
Maternal-HCD leads to the acquirement of senescence-like features in sAT of newborn mice **A.** Effects of maternal normal (m-NCD) and high calorie diet (m-HCD) on sAT weight (*left panel*) and morphology after histochemical H&E staining (*right panel*) in newborn mice. **B.**-**D.** Impact of m-NCD and m-HCD on β-galactosidase activity **B.**, inflammatory **C.** and fibrosis markers **D.** in sAT of newborn mice. β-Actin served as loading control. All graphs are expressed as mean ±S.D. (*n* = 6 female offspring mice/group; *p < 0.05 *vs* m-NCD).

To assess the effects of maternal HCD (m-HCD) in the offspring, we fed mothers with HCD prior and during gestation and lactation. sAT was then analysed in newborns at the end of lactation. When compared to the group of pups born from mothers fed with NCD (m-NCD), m-HCD pups showed sAT expansion that was associated with the onset of SASP. In particular, m-HCD caused an increase of sAT weight and adipocyte size (Figure 2A) in association with increased positivity to SA-β-Gal stain (Figure 2B) and increased levels of senescence molecular markers including H2A.Xp, p21^Waf1^, S6K phosphorylation (Figure 2B) and inflammatory cytokines (Figure 2C). Also, the protein hormone adiponectin (ADIPOQ), whose level is inversely correlated with fat percentage [19], was significantly decreased (Figure 2C). In parallel, no variations of fibrosis markers were observed (Figure 2D). These results demonstrated that m-HCD caused SASP in sAT of offspring mice.

**Figure 3:**
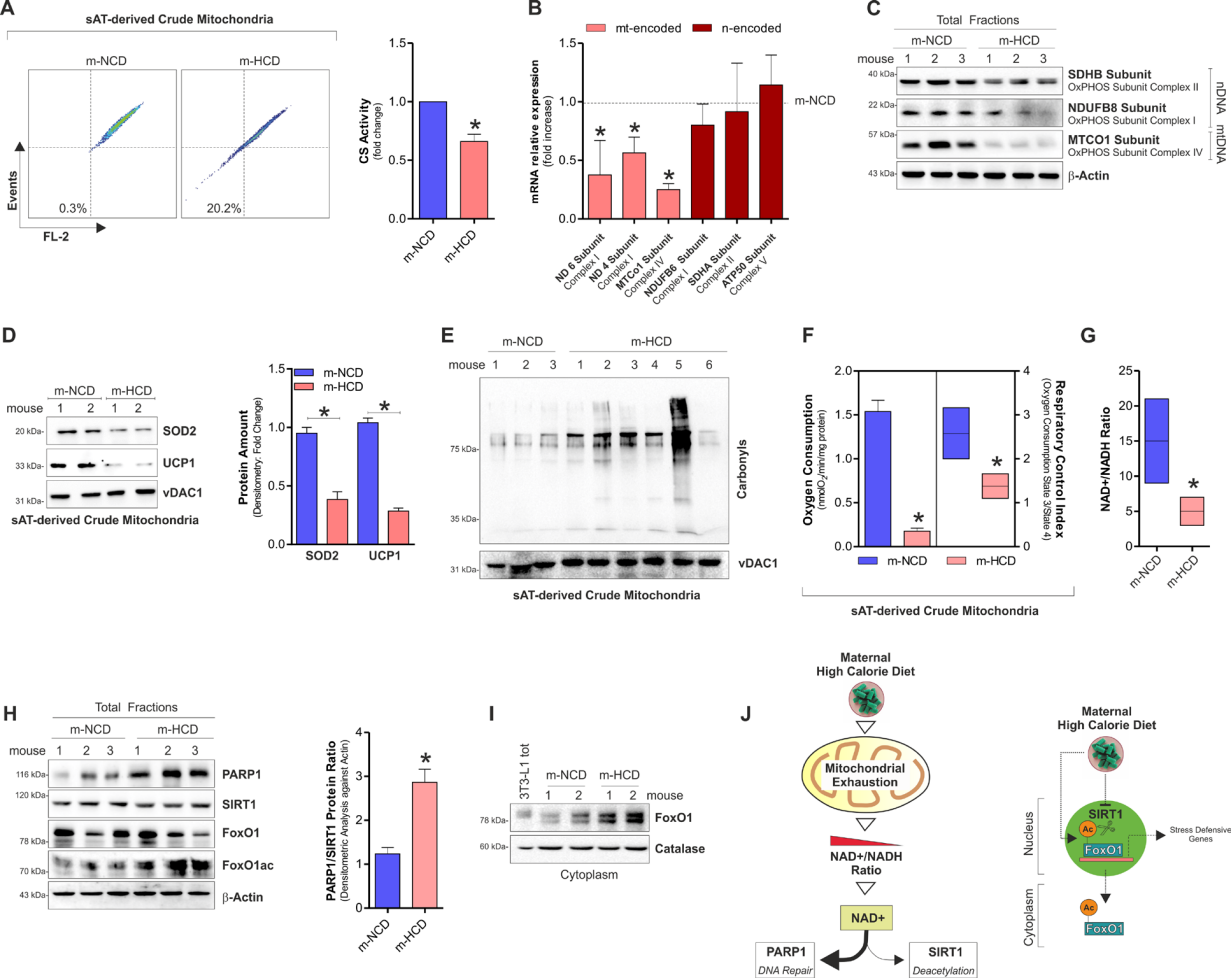
Maternal-HCD induces NAD^+^ decrease, mitochondrial dysfunction and SASP in sAT of newborn mice **A.** Cytofluorimetric analyses of mitochondrial membrane potential (*left panel*) and enzymatic determination of citrate synthase activity (CS) (*right panel*) in sAT of newborn mice. **B.**, **C.** mRNA expression of mtDNA-encoded and nDNA-encoded genes **B.** and protein levels **C.** of mitochondrial OxPHOS subunits in sAT of newborn mice. **D.** SOD2 and UCP1 protein levels (*left panel*) and their densitometric analyses (*right panel*) in sAT crude mitochondria of newborn mice. **E.** Protein carbonylation in sAT crude mitochondria of newborn mice. **F.** Oxygen consumption and respiratory control index in sAT crude mitochondria of newborn mice. **G.** NAD^+^/NADH ratio measured in total homogenates from sAT of newborn mice. **H.** Protein levels of PARP1, SIRT1, FoxO1 and its acetylated form (*left panel*), and densitometric analysis of PARP1/SIRT1 protein ratio (*right panel*) detected in sAT of newborn mice. **I.** Cytoplasmic levels of FoxO1 in sAT of newborn mice. 3T3-L1 lysates served as control of FoxO1 antibody specificity. **J.** Schematic representation of the m-HCD impact on mitochondrial adaptations and molecular responses in newborn mice. β-Actin and vDAC1 served as loading control. For figures D and E the same loading control was reported. All data are expressed as mean ±S.D. (*n* = 6 female newborn mice/group; *p < 0.05 *vs* m-NCD).

Successively, by analysing crude mitochondria we found that m-HCD significantly reduced transmembrane potential and citrate synthase activity (Figure 3A). m-HCD also reduced mtDNA gene transcription (Figure 3B), the level of oxidative phosphorylation subunit complexes (Figure 3C) as well as the level of the antioxidant SOD2 and the thermogenic/antioxidant protein UCP1 (Figure 3D). Concomitantly, m-HCD caused oxidative damage to mitochondrial proteins as a higher content of carbonylated residues was detected (Figure 3E). Mitochondrial bioenergetics profile of m-HCD pups was characterized by lower oxygen consumption, respiratory control index (Figure 3F) and NAD^+^/NADH ratio (Figure 3G).

PARP1 and SIRT1 are enzymes that compete for the common NAD^+^ substrate and, in case of scarce NAD+ availability, deacetylase activity of SIRT1 might result impaired [11]. We found that, along with NAD^+^ depletion, m-HCD caused PARP1 increase, whereas SIRT1 protein mainly remained unchanged, highlighting an imbalanced ratio between these enzymes (Figure 3H). FoxO1 represents a substrate of SIRT1, which promotes its activation *via* deacetylation and redistribution from the cytoplasm to the nucleus [20]. The analysis of acetylated/inactivated and cytoplasmic level of FoxO1 confirmed a diminished NAD^+^ availability for SIRT1 activity in m-HCD with respect to m-NCD offspring. Actually, FoxO1 was highly acetylated (Figure 3H) and accumulated within cytoplasmic compartment (Figure 3I). Hence, it can be hypothesized that the m-HCD, by triggering mitochondrial exhaustion, limited NAD^+^ availability thus affecting NAD^+^-dependent enzymes (Figure 3J).

**Figure 4:**
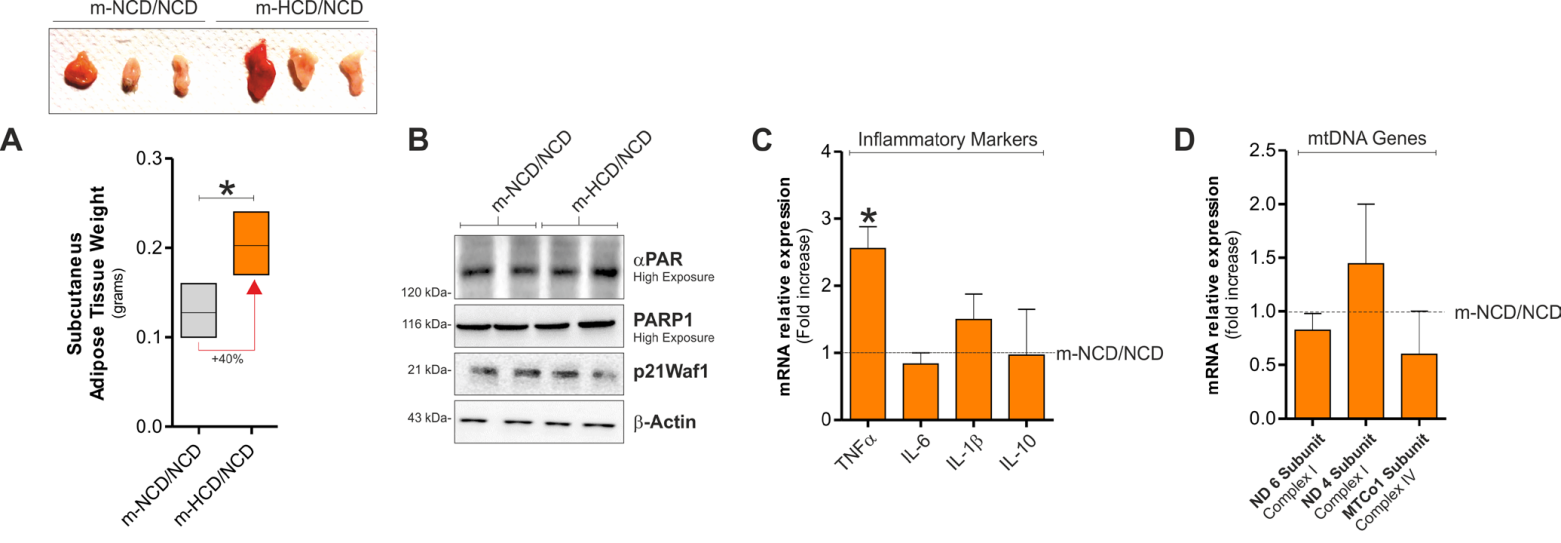
Weaning with NCD recovered senescence features and mitochondrial alteration in the adulthood **A.** Effects of weaning with NCD on sAT weight in m-NCD and m-HCD mice. **B.** PARP1, αPAR and p21^Waf1^ protein levels in m-NCD/NCD and m-HCD/NCD mice. **C.**, **D.** Inflammatory cytokines **C.** and mtDNA-encoded gene expression **D.** in sAT of m-NCD/NCD and m-HCD/NCD mice. β-Actin served as loading control. All graphs are expressed as mean ±S.D. (*n* = 4 female offspring mice/group; *p < 0.05 *vs* m-NCD/NCD).

We then moved at analysing sAT of m-HCD mice after weaning with NCD up to six weeks from the end of lactation. As reported in Figure 4, even though m-HCD mice still showed enlarged fat mass (Figure 4A), weaning with NCD restored the level of DNA repair-related proteins such as PARP1, PARP1 substrates and p21 (Figure 4B), the majority of the inflammatory cytokines (Figure 4C) as well as mtDNA-encoded genes (Figure 4D).

### Niacin limits the occurrence of SASP in offspring mice

**Figure 5:**
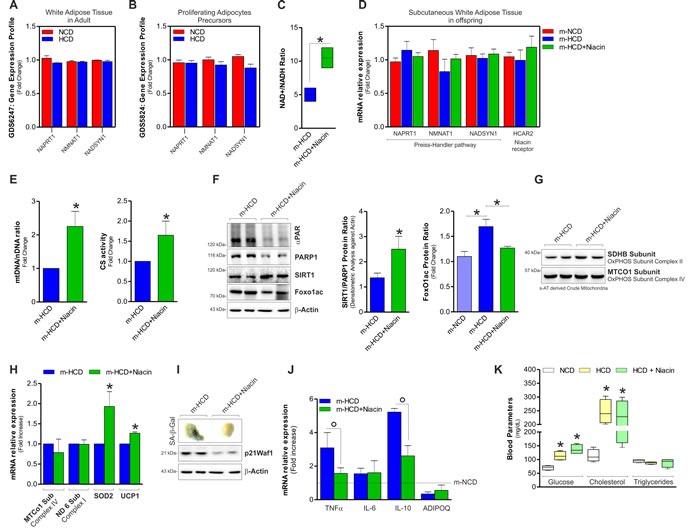
Niacin prevents m-HCD driving senescent phenotype in sAT of newborn mice **A.**, **B.** Analyses of data from the publicly available GEO data set revealed a detectable expression of genes related to NAD^+^ synthesis from niacin (Preiss-Handler pathway) in adult white adipose tissue (ID: GDS6247) **A.** and proliferating adipocytes precursors (ID: GDS5824) **B.** of mice fed with NCD or HCD. **C.** NAD^+^/NADH ratio in sAT of newborn mice supplemented or not with niacin. **D.** Measurement of the mRNA expression of Preiss-Handler pathway and HCAR2 genes in sAT of newborn mice supplemented or not with niacin. **E.** Fold change in mtDNA/nDNA ratio (*left panel*) and citrate synthase (CS) activity (*right panel*) detected in sAT of newborn mice supplemented or not with niacin. **F.** Protein levels of PARP1, SIRT1 and acetylated FoxO1 form in sAT of newborn mice supplemented or not with niacin (*left panel*), densitometric analysis of PARP1/SIRT1 (*central panel*) and acetyl FoxO1 protein ratio (*right panel*). **G.**, **H.** Protein levels of mitochondrial OxPHOS subunits detected on crude mitochondria **G.**, mtDNA-encoded and nDNA-encoded genes expression **H.** in sAT of newborn mice born from mothers supplemented or not with niacin. **I.**, **J.** Analysis of senescence **I.** and inflammatory **J.** markers in sAT of newborn mice supplemented or not with niacin. **K.** Glucose, cholesterol and triglyceride levels detected in blood of mothers 12 weeks after NCD and HCD. β-Actin served as loading control. All data are expressed as mean ±S.D. (*n* = 6 female newborn mice/group; *p < 0.05 *vs* m-HCD).

Avoiding NAD^+^ drop by pharmacological or dietary strategies limits senescent signatures and improves mitochondrial activity [9, 10]. Niacin is used by the cells to synthetize NAD^+^ and it has been used as nutritional supplement also in human even during gestation [21]. Figure 5A and 5B show the GEO data analysis of the expression of genes involved in NAD^+^ synthesis from niacin (Preiss-Handler pathway; i.e. Naprt1, Nmnat1, Nadsyn1) upon feeding with HCD in mice. This analysis indicated that Naprt1, Nmnat1 and Nadsyn1 are expressed in adult mice both in white adipose tissue and proliferating stromal vascular adipocyte precursors (GDS6247; GDS5824) [22, 23]. Moreover, their expression was not affected by HCD (Figure 5A and 5B). These data strongly indicated the ability of sAT in synthetizing NAD^+^ from niacin. We thus attempted at elevating NAD^+^ level in pups by dietary supplementing HCD-fed mothers with niacin. Figure 5C shows that in pups niacin elevated NAD^+^/NADH ratio without altering the expression of genes related to NAD^+^ synthesis (Figure 5D).

The increase of NAD^+^ level was associated with an enhancement of mitochondrial mass. Actually, mtDNA/nDNA ratio and citrate synthase activity resulted increased in niacin-treated m-HCD pups with respect to niacin-untreated m-HCD pups (Figure 5E). Niacin prevented the imbalance between PARP1 and SIRT1 detected in m-HCD (Figure 5F). Indeed, a reduced acetylated/inactive form of FoxO1 was observed (Figure 5F), indicating an efficient NAD^+^ replenishment for the deacetylase activity of SIRT1. In parallel, a reduced presence of poly(ADP-ribose) polymers on PARP1 (αPAR) was observed. The analysis of the expression of oxidative phosphorylation subunit complexes revealed that mtDNA-encoded proteins remained affected, while the nuclear-encoded SDHB was slightly recovered (Figure 5G, 5H). The same results were obtained in sAT of HCD-fed mothers (data not shown). As we observed a significant decrease of the antioxidant proteins SOD2 and UCP1, which are also well-known downstream targets of FoxO1 [24], we looked at their mRNA expression levels upon niacin supplementation and we found them significantly recovered (Figure 5H). Notably, niacin prevented SASP in sAT of m-HCD pups as demonstrated by reduced SA-β-Gal^+^, p21^Waf1^ protein level (Figure 5I) and decreased mRNA expression of the pro-inflammatory cytokines TNFα and IL-10 (Figure 5J). On the contrary, IL-6 did not turn back to basal level upon niacin treatment (Figure 5J). Importantly, the beneficial effects of niacin on offspring sAT did not depend on the amelioration of overall systemic metabolism in mothers. As reported in Figure 5K, glycaemia and cholesterolemia, that resulted significantly affected in HCD fed-mothers, did not return back to basal level upon niacin supplementation.

Niacin may increase NAD^+^ level also indirectly *via* a signalling pathway involving the recruitment of the so-called “niacin receptors” and the downstream up-regulation of adiponectin expression [25]. We found that niacin supplementation did not affect the expression of the niacin receptor HCAR2 (Figure 5D) and failed in counteracting adiponectin decrease in m-HCD pups (Figure 5J). This indicated a lack of the involvement of such signalling pathway in the beneficial effects of niacin on sAT.

## DISCUSSION

Mitochondria are cellular bioenergetics hubs that manage nutrient flux according to cellular and tissue needs. Chronic excess of nutrients, particularly in the early stage of life, inevitably compromises mitochondrial function and profoundly affects health and life span. In a previous work we demonstrated that m-HCD irreversibly disrupted brown adipose tissue (BAT) function in offspring mice [16]. Herein, we demonstrated that, both in mothers and offspring mice, m-HCD induced mitochondrial dysfunction in sAT. However, we also found that, differently to mothers, sAT of offspring mice displayed a senescent and inflammatory phenotype, which was associated with a significant NAD^+^/NADH drop. In contrast to BAT, sAT dysfunction can be partially recovered by feeding with NCD. The significance of almost fully recovered sAT dysfunction after NCD could highlight specific functional differences with respect to BAT. Physiologically, BAT participates in thermogenesis program having mitochondria equipment arranged for maintaining uncoupled oxidative phosphorylation. Under thermoneutrality mitochondria of BAT preferentially use glucose as elective substrate, whereas upon cold exposure substrate utilization is shifted towards lipids. Selective fuel choice by mitochondria assures tissue functionality by an intricate network of metabolic and signalling pathways [8]. A persistence in dietary fat overload during development may force BAT mitochondria to acquire white-like functions (whitening) thus failing in its physiological functionality as thermogenic tissue [26]. Differently to BAT mitochondria, sAT mitochondria are well equipped for ATP synthesis (coupled respiration). Due to low activity of carnitine palmitoyltransferase 1 in the inner mitochondrial membrane, oxidation of fatty acids is relatively slow and fatty acids are directed towards esterification into triglycerides [27]. This feature confers a strong capability of sAT to buffer fatty acid flux occurring after high fat meals. Notably, sAT also displays the capability to undergo brown-like changes upon cold stimuli or nutrient scarcity [28]. Indeed, these conditions increase mitochondrial oxidative capacity and promote energy dissipation (browning) [29]. Secondarily, sAT is characterized by high abundance of proliferating mesenchymal stem cells [30], which may be promptly engaged upon normal chow diet and rejuvenate the tissue. If compared to BAT, such sAT plasticity might at least partially justify the recovery from MiDAS phenotype upon NCD. This suggests that BAT impairment might play a major role in the onset of metabolic disturbances in offspring adulthood. However, given the crucial nutrient buffering role and intrinsic capacity of senescent white adipocytes to produce pro-inflammatory cytokines [31], the persistence of HCD after weaning might predispose to the onset of typical age-related diseases. Actually, a sAT dysregulation consisting in adipocyte hypertrophy accompanied by inflammation has been proposed as the major susceptibility factor for later development of type 2 diabetes [32]. Interestingly, pups weaned with NCD lost senescent phenotype and underwent a partial recovery of cytokine production. This suggests that the detrimental effects of maternal HCD on sAT could be at least in part mitigated in the adulthood lowering the risk of developing metabolic diseases.

sAT of mothers and pups significantly differs in the quantity and proliferation capacity of adipocytes precursors, which are higher in newborns with respect to adults [33]. Mitochondrial dysfunction and decrease of NAD^+^/NADH have been found to induce senescence in proliferating cells. Interestingly, pyruvate supplementation improved NAD^+^/NADH ratio and counteracted the onset of senescence phenotype [9]. Similarly, NAD^+^ precursor nicotinamide riboside was able to rejuvenate muscle and neural stem cells in mice. In the same way, NAD^+^ restoration *via* overexpression of enzymes that control mitochondrial NAD^+^ levels was found to rejuvenate aged somatic cells and extend the lifespan of human mesenchymal stem cells by delaying replicative senescence [10, 34].

In order to unravel whether NAD^+^/NADH reduction was the triggering factor of sAT dysfunction also in our experimental model, we attempted at counteracting HCD-mediated NAD^+^ depletion by supplementing niacin in the mother prior and during gestation and lactation. A recent work showed that nicotinammide riboside supplementation affects adipose tissue inflammation beneficially and raises antioxidant enzymes levels in mice fed with a mild obesogenic diet [35]. Sounding evidence have demonstrated that, similarly to other NAD^+^ booster, such as NMN and nicotinammide riboside, niacin is strongly effective in increasing tissue NAD^+^ level [36]. Niacin can efficiently replenish NAD^+^ both directly *via* its synthesis through the cytosolic Preiss-Handler pathway and indirectly through a signalling pathway involving the recruitment of niacin receptors and up-regulation of adiponectin secretion [25]. Through niacin treatment we significantly avoided NAD^+^ decrease induced by HCD and abrogated SASP in sAT of pups. We suggest that in sAT the elevation of NAD^+^ by niacin has to be mainly ascribed to its neo-synthesis. Indeed, the enzymes of NAD^+^ synthesis are adequately expressed and their mRNA levels are not affected by HCD. Moreover, the unresponsiveness of adiponectin to niacin treatment let us hypothesizing that NAD-dependent signalling pathway involving niacin receptors do not take centre place in buffering NAD^+^ decrease imposed by HCD.

It is also possible that niacin could mediate an improvement in mother's metabolism that may restore offsprings’ sAT dysfunction secondarily. Actually, the metabolic benefits of niacin supplementation are due to its ability to reduce lipidemia by blocking lipolysis in adipose tissue and liver [37]. It has been however demonstrated that niacin treatment has lipid-lowering effects during NCD, while it is not able to reduce lipidemia in mice fed with HCD [25]. Accordingly, our data show that niacin failed in decreasing blood cholesterol levels in mothers. This reinforces the idea that the beneficial effects of niacin on offspring sAT mainly rely on its NAD replenishment activity.

**Figure 6:**
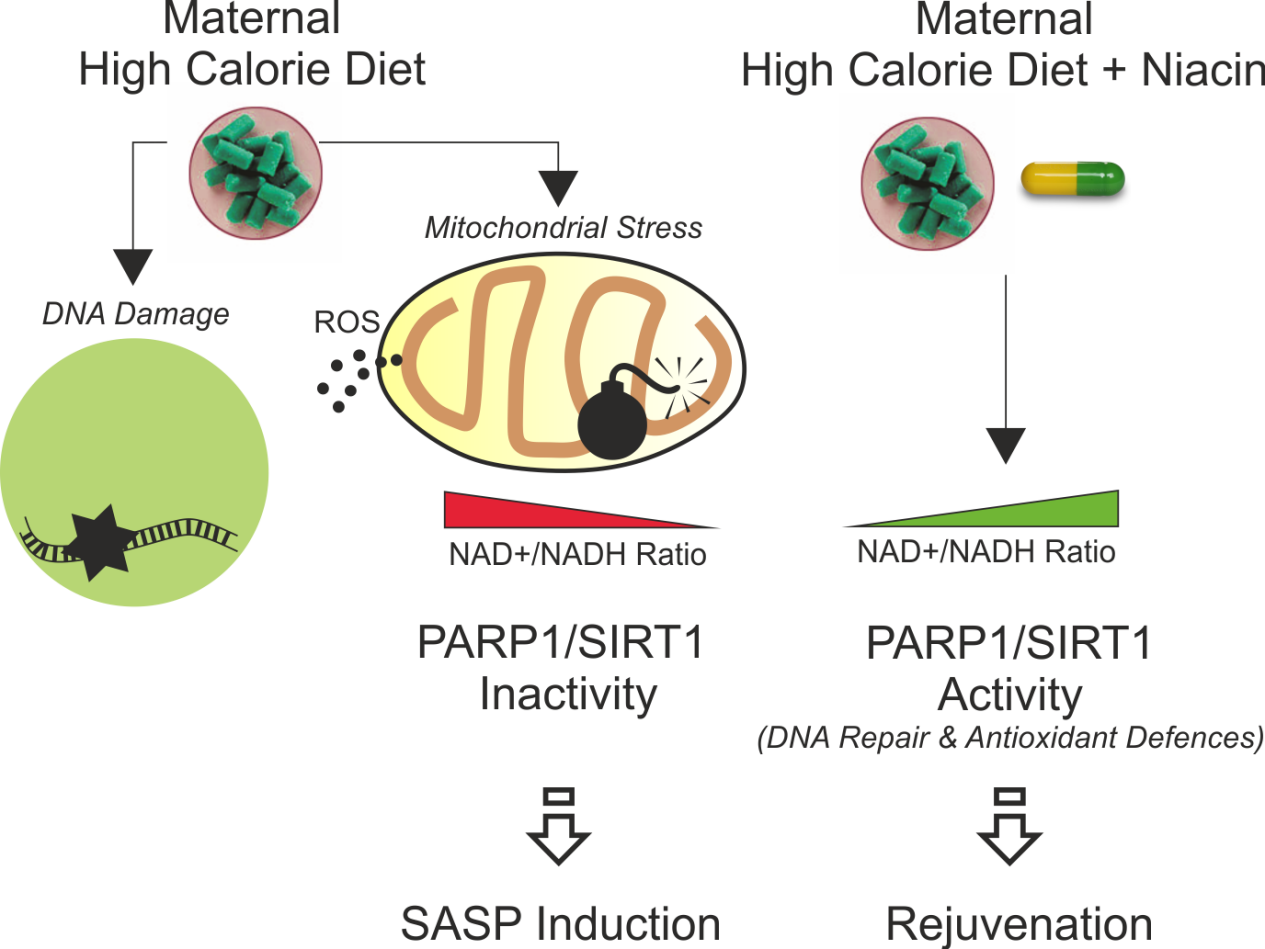
Schematic representation of the impact of maternal high calorie diet in offspring subcutaneous adipose tissue and rejuvenating effects of niacin

Contrarily to vAT expansion, sAT expansion can be highly protective by maintaining overall body metabolic homeostasis. Deterioration of sAT inevitably results in metabolic complications and accelerates aging. Actually, sAT represents the main storage and buffering system against excess of nutrients, avoiding visceral and ectopic fat accumulation [6]. Adipose tissue expansion can occur through an enlargement in adipocyte size or an increase in adipocyte number. Differentiated adipocytes are post-mitotic; therefore, hyperplasia represents an increase in proliferating rate of adipocyte precursors. Our findings suggest that HCD initially induced mitochondrial hyperactivity and ROS production that prompt genotoxic damage, leading to proliferative potential limitation in adipocyte precursors (Figure 6). Indeed, sAT develops between embryonic days 14 and 18. As consequence, limiting its expansion through proliferation in the early phases of life could be metabolically detrimental in the later phase of life. Furthermore, the persistence of high calorie diet forced hypertrophic and consequentially hypersecretory phenotype (hallmarks of senescence) in cell cycle blocked preadipocytes. Differently to subcutaneous fat, visceral fat develops postnatally, justifying the induction of a senescent phenotype in visceral fat of adult mice upon HCD [38]. Indeed, even if HCD significantly induced sAT expansion in adult mothers, we did not reveal any SASP in this adipose depot. This is consistent with the aspect that hypertrophy is the main response occurring in differentiated adipocytes of sAT in adult mice upon HCD. On the contrary, being sAT of pups enriched in proliferating adipocytes precursors, in this adipose depot m-HCD induces senescent signatures that nicely resemble to mitochondrial dysfunction-associated senescence (MiDAS) [9]. In line with this assumption, we found that IL-6 production, which is normally increased in senescence associated with genotoxic stress [39], is only slightly affected by m-HCD and not inhibited by niacin.

The evidence that in m-HCD group the markers of MiDAS, such as TNFα, IL-1β and IL-10, are highly increased and down-regulated by niacin consumption further validates the hypothesis of MiDAS occurrence. Our results are in line with other studies describing the effectiveness of NAD^+^ replenishment in preventing cellular and tissue senescence by mitochondrial activity restoration [9–11].

In our model, the rejuvenating action of niacin-derived NAD^+^ was accompanied by an improvement only of mitochondrial metabolic enzymes encoded at nuclear level. Indeed, mtDNA-encoded proteins remained affected upon niacin supplementation. Also based on our results showing an increased SIRT1 and PARP1 activity and inhibited p21 accumulation in m-HCD pups supplemented with niacin, we can suggest that niacin-derived NAD^+^ only acts at the nuclear level. This assumption is nicely supported by a recent paper demonstrating that niacin supplementation efficiently promotes nuclear DNA repair in X-irradiated human peripheral blood cells *via* the enhancement of SIRT1 activity [40].

A key co-regulatory mechanism between nuclear SIRT1 and PARP1 enzymatic activity is the utilization of NAD+. PARP1 activation upon DNA stress causes a depletion in NAD + levels in nuclei that inhibits SIRT1 deacetylating activity (PARP1/SIRT1 metabolic imbalance) [11]. In m-HCD, dysfunctional mitochondria further limit NAD+ availability in DNA damaged cells, strongly affecting DNA repair and deacetylation of SIRT1 substrates. The persistence of this metabolic stress drives a senescence phenotype in sAT of newborns. Niacin likely prevents SASP induction in offspring mice by promoting the SIRT1/FoxO1-mediated expression of antioxidant enzymes and PARP1-mediated DNA repair (Figure 6). Accordingly, we found that the level of acetylated FoxO1, a well-known substrate of SIRT1, is decreased upon niacin treatment and this correlates with a restored expression of FoxO1-downstream antioxidant targets, i.e. SOD2 and UCP1. Moreover, in previous works we demonstrated that FoxO1 plays a key role in triggering lipolysis in adipocytes by promoting lipases expression, hence, we cannot exclude that niacin-mediated recovery of FoxO1 activity positively impacts on adipose tissue lipolysis in m-HCD pups [41, 42].

Collectively, our data give further support to the idea that NAD^+^ plays a critical role in assuring overall body metabolic homeostasis by counteracting sAT dysfunction and SASP induction. Dietary strategies aimed at enhancing NAD^+^ levels during gestational diabetes in obese or overweight mothers can hence be fruitful for decreasing the risk of developing metabolic diseases in the offspring during life.

## MATERIALS AND METHODS

### Mice and treatments

Mouse experimentation was conducted in accordance with accepted standard of humane animal care after the approval by relevant national (Ministry of Health) and local (Institutional Animal Care and Use Committee, Tor Vergata University) committees. Sample size was calculated by using Sample Size Calculator & Power Analyses Software (http://www.statisticalsolutions.net/pss_calc.php) to reject the null hypothesis of no difference. A total of twelve C57BL/6J adult (2 months-age-old) female mice (purchased from Harlan Laboratories S.r.l., Urbino, Italy) were randomly divided in three groups: normal calorie diet (NCD) group (3.85 kcal/g among which 10% kcal from fat, 20% from protein and 70% from carbohydrate), high calorie diet (HCD) group (5.24 kcal/g among which 60% kcal from fat, 20% from protein, and 20% from carbohydrate) and HCD group supplemented with niacin (Sigma-Aldrich, N4126) at the dose of 400 mg/kg [36] in drinking water. Dietary treatments were started 8 weeks before mating and then maintained throughout pregnancy and lactation. Pups born from mothers fed with NCD (m-NCD), with HCD (m-HCD) or with HCD plus niacin (m-HCD + niacin) were fostered by mothers on the same diet (*n* = 6 mice per group) up to weaning and then sacrificed. A group of m-NCD and m-HCD pups was weaned with NCD, which was maintained for 6 weeks, and then sacrificed. Mice were starved overnight (12 h) prior to sacrifice. Mice were maintained with 12 h light/dark cycles and had free access to food and water. Before sacrifice mice were starved for 16 h, weighed and blood collected to perform bioclinical analyses by a KeyLab analyser by using specific assay kits (BPCBioSed, Italy). After cervical dislocation, adipose tissues were explanted and immediately analysed.

### Subcellular fractionation

Cytoplasmic fraction was obtained by using a commercially available kit (NE-PER^®^ extraction kit, according to manufacturer's instructions (Thermo Scientific, Milwaukee, WI, USA). Crude mitochondrial fractions were obtained as previously described [17].

### Gel electrophoresis and Western blot

Crude mitochondrial and total fractions were lysed in RIPA buffer (50 mM Tris-HCl, pH 8.0, 150 mM NaCl, 12 mM deoxycholic acid, 0.5% Nonidet P-40, and protease and phosphatase inhibitors). Proteins were loaded for SDS-PAGE followed by Western blotting. Nitrocellulose membranes were incubated with primary antibodies and successively with the appropriate horseradish peroxidase-conjugated secondary antibodies. Immunoreactive bands were detected by a Fluorchem Imaging System after incubation of the membranes with ECL Selected Western Blotting Detection Reagent (GE Healthcare, Pittsburgh, PA, USA).

### Histochemical analysis and senescence-associated beta-galactosidase activity

To evaluate adipocyte morphology, explanted sAT was fixed in Carnoy's liquid, dehydrated and embedded in paraffin. Successively, paraffin-embedded tissues were cut in 7 mm sections and stained with hematoxylin and eosin (H&E) prior microscope analysis. The sAT SA-β gal staining was carried out as previously reported with minor modifications [41]. Briefly, sAT was fixed in 4% paraformaldehyde for 72 h and then incubated for 4 h at 37°C in a pH 6.0 β-gal staining solution (1 mg/ml 1,5-bromo-4-chloro-3-indolyl β-D-galactopyranoside, 5 mM potassium ferrocyanide, 5 mM potassium ferricyanide, 150 mM NaCl, 2 mM MgCl_2_) and images acquired by a digital camera.

### RT-qPCR analysis

Total RNA was extracted using TRI Reagent^®^ (Sigma-Aldrich). RNA (3 μg) was retro-transcripted by using M-MLV (Promega, Madison, WI). qPCR was performed in triplicate by using validated qPCR primers (BLAST), Ex TAq qPCR Premix, and the Real-Time PCR LightCycler II (Roche Diagnostics, Indianapolis, IN) as described by Lettieri Barbato et al. [42]. mRNA levels were normalized to actin mRNA, and the relative mRNA levels were determined through the 2^−ΔΔ^Ct method.

### Determination of mtDNA/nDNA ratio

Samples were digested with proteinase K and total DNA was extracted through phenol-chloroform and ethanol precipitation method. mtDNA copy number was analysed by qPCR as described by Yatsuga and Suomalainen [43]. 12S rRNA gene primers and actin gene primers were used for mtDNA and nDNA. 12S rRNA levels were normalized to nuclear actin gene, and the relative mtDNA levels were determined through the 2^−ΔΔ^Ct method.

### Assay of mitochondrial membrane potential

Crude mitochondria were resuspended in mitochondrial activity buffer (70 mM sucrose, 220 mM mannitol, 2 mM HEPES buffer, 5 mM magnesium chloride, 5 mM potassium phosphate, 1 mM EDTA, 5 mM succinic acid, and 0.1% fatty acid free bovine serum albumin, pH 7.4) and incubated with 250 nM MitoTracker Red CMX ROS and 250 nM Mitotracker Green (Life Technologies Ltd.) for 30 min, 37°C. Afterwards, analysis was performed in stained mitochondria through a FACScalibur instrument (Beckton and Dickinson, San Jose`, CA, USA). Mitochondrial population was selected by gating particles that were positive to Mitotracker Green staining. MitoTracker Red fluorescence intensity was measured in 200,000 mitochondria.

### Oxygen consumption and respiratory control index

Oxygen consumption was determined in crude mitochondria using the Oxygraph Plus oxygen electrode system (Hansatech Instruments Ltd., Norfolk, UK). Crude mitochondria were resuspended in an appropriate mitochondrial activity buffer (70 mM sucrose, 220 mM mannitol, 2 mM HEPES buffer, 5 mM magnesium chloride, 5 mM potassium phosphate, 1 mM EDTA, 5 mM succinic acid, and 0.1% fatty acid free bovine serum albumin, pH 7.4). Next, oxygen consumption was monitored at 37°C, for 6 min. Respiratory control index was expressed as ratio between ADP-activated mitochondrial respiration (state 3) and steady state (state 4) of respiration. Oxygen consumption was normalized for protein concentration.

### NAD^+^/NADH ratio

NAD^+^/NADH ratio was detected in total homogenates by using NAD^+^/NADH Quantitation Kit according to manufacturer's instruction (Sigma-Aldrich). All values were normalized for protein concentration.

### Citrate synthase activity and protein carbonyls detection

Citrate synthase activity was measured by using Citrate Synthase Activity Assay Kit (Abcam, Cambridge, UK). Carbonylated proteins were detected by using the OxyBlot Protein Oxidation Detection Kit (EMD Millipore). All values were normalized for total protein amount.

### Bioinformatics analysis

sAT microarray data from young, aged or aged rats treated with calorie restriction were analysed for transcript expression using gene set enrichment analysis. Raw microarray data are publically available on GEO under the accession numbers GDS3102. List of genes for bioinformatics analysis was: NDUFA2, NDUFB6, SDHA, SDHB, UQCRC1, UQCRC2, COX5A, COX6C, ATP5B, ATP5O, ND1, ND2, ND3 (for OxPHOS Subunits), IL6, TNFa, IL10, IL1b (for inflammatory cytokines) and ACO2, CS, IDH2, MDH2 (for TCA-related enzymes). Microarray data of sAT and proliferating adipocytes precursors from mice fed with normal diet or high calorie diet are publically available on GEO under the accession numbers GDS6247 and GDS5824 respectively. Gene expression profile analysis was reported as fold change using GraphPad Prism software.

### Statistical analysis

The results are presented as means ± S.D. Statistical analyses were carried out by ANOVA followed by the post Student-Newman-Keuls. Differences were considered to be significant at *p* < 0.05.

## Abbreviations

ADIPOQ: adiponectin
BAT: brown adipose tissue
FoxO1: forkhead box protein O1
IL-1β, IL-6, IL-10: Interleukin-1β, -6, -10
HCD: high calorie diet
m-HCD: maternal- high calorie diet
m-NCD: maternal-normal calorie diet
mtDNA: mitochondrial DNA
MiDAS: mitochondrial dysfunction-associated senescence
NAD: nicotinamide adenine dinucleotide
NCD: normal calorie diet
nDNA: nuclear DNA
OxPHOS: oxidative phosphorylation
PARP1: poly [ADP-ribose] polymerase 1
SA-β-Gal: senescence-associated beta-galactosidase
ROS: reactive oxygen species
SASP: senescence-associated secretory phenotype
sAT: subcutaneous adipose tissue
SIRT1: sirtuin 1
SOD2: superoxide dismutase 2, mitochondrial
TNFα: Tumor necrosis factor alpha
TCA: tricarboxylic acid cycle
UCP1: uncoupling protein 1
vAT: visceral adipose tissue
